# A New Recombinant Multiepitope Chimeric Protein of *Leptospira interrogans* Is a Promising Marker for the Serodiagnosis of Leptospirosis

**DOI:** 10.3390/tropicalmed7110362

**Published:** 2022-11-09

**Authors:** Luis G. V. Fernandes, Kátia E. S. Avelar, Eliete C. Romero, Marcos B. Heinemann, Karin Kirchgatter, Ana L. T. O. Nascimento

**Affiliations:** 1 Laboratório de Desenvolvimento de Vacinas, Instituto Butantan, Avenida Vital Brazil, 1500, Sao Paulo 05503-900, SP, Brazil; 2 Laboratório de Referência Nacional para Leptospirose, Instituto Oswaldo Cruz—Fiocruz, Avenida Brasil, 4365, Manguinhos, Rio de Janeiro 21040-900, RJ, Brazil; 3 Centro de Bacteriología, Instituto Adolfo Lutz, Sao Paulo 01246-902, SP, Brazil; 4 Laboratório de Zoonoses Bacterianas do VPS, Faculdade de Medicina Veterinária e Zootecnia, USP, Sao Paulo 05508-270, SP, Brazil; 5 Laboratório de Bioquímica e Biologia Molecular, Instituto Pasteur, Sao Paulo 01027-000, SP, Brazil; 6 Programa de Pós Graduação em Medicina Tropical, Faculdade de Medicina, Universidade de São Paulo, Sao Paulo 05403-000, SP, Brazil

**Keywords:** *Leptospira*, leptospirosis, chimeric protein, diagnosis, MAT

## Abstract

The zoonotic disease leptospirosis is caused by pathogenic species of the genus *Leptospira* and was recently included in the list of Neglected Diseases by the World Health Organization. Leptospirosis burden is estimated to have over a million human cases and cause 60 thousand deaths annually, in addition to its economic impact and veterinary concern. The microscopic agglutination test (MAT), recommended by the World Health Organization, exhibits reduced sensitivity at the beginning of the disease, in addition to being technically difficult. New recombinant antigens are being pursued for rapid and specific serodiagnostic tests, especially in the initial phase of the disease, and chimeric multiepitope proteins are a strategy with a great potential to be implemented in serology. Based on previous subproteomic results, we designed a synthetic construct comprising 10 conserved leptospiral surface antigens, and the recombinant protein was purified and evaluated regarding its diagnostic potential. The protein termed rChi2 was recognized by antibodies in serum from patients both at the onset (MAT−) and in the convalescent (MAT+) phase in 75 and 82% of responders, respectively. In addition, rChi2 immunization in hamsters elicited a strong humoral response, and anti-rChi2 antibodies recognized several immobilized intact *Leptospira* species, validating its potential as an early, broad, and cross-reactive diagnostic test.

## 1. Introduction

Leptospirosis is a widespread zoonosis caused by pathogenic species of the genus *Leptospira* [1]. Humans and animals can be infected through intact sodden or damaged skin or mucosa. The disease is characterized by a wide range of nonspecific symptoms such as fever, chills, headache, and myalgia. Severe forms with potentially fatal conditions are known as Weil’s disease, corresponding to 5–15% of reported cases, and even more serious cases of leptospirosis-associated pulmonary hemorrhage syndrome (LPHS) can occur, which has a fatality rate of more than 50%. Humans are considered accidental and terminal hosts in the chain of transmission [2,3].

Annually, leptospirosis accounts for an estimated incidence of over one million cases, resulting in approximately 60,000 deaths [3]. The disease has arisen as a health threat in new settings due to globalization and climate change, and outbreaks can be triggered by disasters and extreme weather events [4]. Mortality and incidence statistics are believed to be underestimated because of a multitude of factors, including a lack of surveillance, rapid diagnostic tests and notification in countries with a high disease incidence [5,6].

Diagnosis of leptospirosis in the early stages remains challenging; the most frequently used diagnostic tool for leptospirosis is the reference microagglutination test (MAT), which displays low sensitivity at the onset of the disease, since this method relies on the detection of antibodies mainly against leptospiral LPS. This test requires significant expertise from its users and a large and representative collection of live and routinely maintained cultures of leptospires [1,2]. Direct dark-field microscopy visualization of blood from suspected patients can also be used for early diagnosis during the leptospiremia phase, but it is subject to misinterpretation [7,8].

A diverse set of leptospiral recombinant proteins have been proposed as antigens for ELISA protocols [9,10,11,12,13,14]. Conserved outer membrane proteins (OMPs) among pathogenic *Leptospira* spp. are interesting candidates for composing a diagnostic test for leptospirosis. In addition, it has been shown that the use of several antigens can increase detection sensitivity [12,15], indicating that leptospiral chimeric proteins are an attractive approach [16,17].

Many studies have been published investigating the proteome and secretome of pathogenic strains of *Leptospira* [18,19,20,21]. One such study, performed by Zeng et al. [22], aimed to identify potential vaccine and diagnostic antigen or virulence-determinant candidates against leptospirosis through subproteomic techniques. The authors identified core and unique surface-exposed proteins among several leptospiral strains; with peptide identification and conservation analysis taken together, 10 highly conserved surface-exposed or subsurface proteins were identified, which could be the most suitable vaccine and/or diagnosis candidates to be tested.

Taking advantage of the previous identification of 10 core surface proteins among epidemiologically relevant leptospiral strains [22], we designed a novel chimeric construct containing peptides derived from the orthologs of these proteins in the *L. interrogans* serovar Copenhageni strain Fiocruz L1-130 (LIC10123, LIC13059, LIC13050, LIC12615, LIC11636, LipL32, LIC11122, LIC11089, LIC10713, and LIC13434) and evaluated whether the purified recombinant protein could be recognized by antibodies of leptospirosis patients at the onset or in the convalescent phase.

## 2. Materials and Methods

### 2.1. Bacteria and Serum Samples

The pathogenic *L. interrogans* serovar Copenhageni strain FIOCRUZ L1-130, the *L. kirschneri* serovar Cynoptery strain 3522C, the *L. santarosai* serovar Shermani strain 1342 K, the *L. noguchii* serovar Panama strain CZ 214, the *L. borgpetersenii* serovar Castellonis strain Castellon 3, and the saprophytic *L. biflexa* serovar Patoc strain Patoc1 were cultured at 28 °C under aerobic conditions in liquid Ellinghausen–McCullough–Johnson–Harris (EMJH) medium (Difco, BD, Franklin Lakes, NJ, USA) with 10% (*v*/*v*) *Leptospira* enrichment EMJH (Difco). Paired human serum samples at the onset (n = 36, MAT-negative) and in the convalescent phase (n = 36, MAT-positive) from patients with confirmed leptospirosis were obtained from the serum collection of the Instituto Adolfo Lutz, Sao Paulo, Brazil, and were kindly donated by Dr. Eliete Romero for research purposes only. An additional set of convalescents (n = 142) and normal human serum (NHS) samples (n = 50) were kindly provided by Dr. Avelar from the Laboratório de Referência Nacional para Leptospirose (LRNL), Fiocruz, Rio de Janeiro.

### 2.2. Microscopic Agglutination Test (MAT)

The MAT was performed as previously described by Faine et al. [7]. Briefly, 22 serovars of *Leptospira* spp. were used as antigens for agglutination: Australis, Autumnalis, Bataviae, Canicola, Castellonis, Celledoni, Copenhageni, Cynopteri, Djasiman, Grippotyphosa, Hardjo, Hebdomadis, Icterohaemorrhagiae, Javanica, Panama, Patoc, Pomona, Pyrogenes, Sejroe, Shermani, Tarassovi, and Wolffi. Leptospires were cultured in EMJH liquid medium at 28 °C. A laboratory-confirmed case of leptospirosis was defined via the demonstration of a 4-fold increase in the microagglutination titer between paired serum samples. MAT was considered negative when the titer was below 100.

### 2.3. Composition and Design of Multiepitope Chimeric Protein

The ten proteins that composed the chimeric protein were selected based on previous comparative subproteome analysis work by Zeng et al. [22]. The amino acid sequences of the selected protein counterparts in *L. interrogans* serovar Copenhageni were obtained from the GenBank database. The ANTIGENIC program in EMBOSS (http://www.bioinformatics.nl/cgi-bin/emboss/antigenic (accessed on 15 June 2015)) and SMART were used to predict B-cell epitopes and putative conserved domains, respectively. Different polypeptide portions were interspaced by a flexible peptide linker (GGGGSGGGGSGGGGS), aiming for the independent folding of each region [16]. The predicted tertiary structure of the recombinant protein was defined using i-TASSER [23] software and visualized using PyMol. The final chimeric amino acid sequence was reverse-translated using an *E. coli* codon usage table in the free Web server http://www.bioinformatics.org/sms2/rev_trans.html (accessed on 20 June 2015). The synthetic gene was manufactured by Invitrogen (http://www.thermofisher.com/invitrogen (accessed on 18 August 2015) and supplied in a T7 promoter-driven 6xHis epitope expression plasmid. The conservation of each protein portion among sequences of the different species of *Leptospira* was performed using BLAST (http://blast.ncbi.nlm.nih.gov/Blast.cgi (accessed on 6 May 2019).

### 2.4. Expression and Purification of Recombinant Chimeric Protein (rChi2)

The recombinant plasmid carrying the chimeric gene was manufactured by Invitrogen (ampicillin resistance) and used to transform *E. coli* BL21 (DE3) cells. The plasmid allows the inclusion of a 6xHis epitope at the N-terminus of the recombinant protein, whose transcription is driven by the T7 promoter. Protein expression was achieved via the inoculation of 5 mL of a saturated culture grown overnight in 200 mL of fresh lysogeny broth (LB) medium containing 50 μg/mL ampicillin. The culture was incubated with continuous shaking at 37 °C to an optical density at 600 nm (OD600 nm) of 1, and the expression of the recombinant protein was induced using 1 mM isopropyl β-d-1-thiogalactopyranoside (IPTG) for 16 h under constant agitation at 18 °C to reduce protein degradation [24]. The cells were recovered via centrifugation, and the resulting pellet was resuspended in sonication buffer (20 mM Tris–HCl (pH 8.0), 200 mM NaCl, 200 mg/mL lysozyme, 2 mM phenylmethylsulfonyl fluoride (PMSF), and 1% Triton X-100) and lysed with the aid of a sonicator tip in an ice bath (Ultrasonic Processor; GE Healthcare Bio-Sciences Corp., Piscataway, NJ, USA). After centrifugation (12,000× *g* for 10 min), the insoluble fraction was resuspended in a buffer containing 20 mM Tris–HCl (pH 8.0), 500 mM NaCl, 20 mM imidazole, and 8 M urea. The recombinant protein was purified through a Ni^2+^-charged chelating fast-flow chromatographic column (GE Healthcare, Buckinghamshire, UK). Buffer containing 20 mM Tris–HCl (pH 8.0), 500 mM NaCl and increasing imidazole concentrations (40 and 60 mM) was used to eliminate loosely bound protein contaminants. Bound recombinant proteins were eluted with buffer containing 1 M imidazole. Different aliquots from the washing and elution steps were analyzed using 12% sodium dodecyl sulfate polyacrylamide gel electrophoresis (SDS-PAGE). Aliquots containing the recombinant protein were mixed and extensively dialyzed against 20 mM Tris–HCl (pH 8) and 500 mM NaCl up to 4 times.

### 2.5. Immunoblotting Assay

The purified recombinant protein was loaded onto 12% SDS-PAGE gels, fractionated and electrotransferred onto nitrocellulose membranes (Hybond ECL; GE Healthcare) on semidry equipment. A solution containing 10% non-fat dry milk in PBS 0.05% Tween 20 (PBS-T) was used to block unspecific sites in the membranes, which were then incubated with hamster serum for 1 h at room temperature. After extensive washing steps, the membranes were incubated with horseradish peroxidase (HRP)-conjugated anti-hamster IgG (1:5000) in PBS-T 10% non-fat dry milk for 1 h. The transferred recombinant protein was also incubated with HRP-conjugated monoclonal anti-His tag antibodies (1:10,000) (Sigma, St. Louis, MO, USA). For the detection of human antibodies against epitopes contained in the chimeric protein in the leptospirosis serum samples, membranes were incubated with pooled MAT-positive, MAT-negative, or NHS samples (1:1000), and then, with HRP-conjugated anti-human IgG (1:10,000) or anti-human IgM (1:10,000) (Sigma). The negative control protein BSA was also included in these experiments. Detection was revealed using Super Signal West Dura Extended Duration Substrate (Thermo Fisher, Waltham, MA, USA).

### 2.6. ELISA for Detection of Antibody against rChi2

ELISA plates were coated with rChi2 recombinant protein (500 ng/well) for 16 h at 4 °C. Wells were washed 3 times with PBS-T, and then, blocked with 200 μL of PBS-T 10% non-fat dry milk for 2 h at 37 °C. After blocking, pooled or individual patients’ paired sera at the onset or in the convalescent phase from 10 patients with confirmed leptospirosis were diluted (1:100 to 1:800) and evaluated for total IgG or IgM against the recombinant protein using horseradish peroxidase (HRP)-conjugated anti-human IgG or IgM antibodies (1:10,000). The reaction was detected by adding 1 mg/mL o-phenylenediamine dihydrochloride (OPD, Sigma-Aldrich) in citrate phosphate buffer (pH 5.0) plus 1 μL/mL H_2_O_2_. The reaction was carried out for 10 min and stopped by the addition of 50 μL of 2 M H_2_SO_4_. The serum samples were previously incubated with 10% *E. coli* whole cell lysate in PBS-T 10% non-fat dry milk solution to prevent the reactivity of anti-*E. coli* antibodies, as described previously [25]. Cutoff values were set at 3 standard deviations above the mean OD492 nm value obtained from NHS (n = 50) against the recombinant protein. The samples with values above the cutoff were considered positive for this experiment. Sera from patients with unrelated febrile illness, including dengue, Chagas’ disease, HIV, and malaria (n = 10 each), were also employed for assessing the specificity of the reactions.

Mouse polyclonal antisera against recombinant proteins whose epitopes are contained in the chimeric protein were also evaluated. Coating and blocking were performed as described above, and the coated protein was incubated with different dilutions of antisera, followed by incubation with HRP-conjugated anti-mouse IgG (1:5000, Sigma) and development with OPD substrate.

### 2.7. Immunogenicity of rChi2 in Hamster Model

Male Syrian hamsters (*Mesocricetus auratus*, 6–8 weeks old, n = 6) were subcutaneously immunized with 50 μg of rChi2 mixed with 12.5% Alhydrogel (2% Al(OH)_3_) as an adjuvant. One booster injection was given after 2 weeks with the same preparation of recombinant protein. The hamsters in the negative control group were injected with Tris-NaCl buffer in 12.5% Alhydrogel. Hamsters received water and food ad libitum and were monitored daily. Hamsters in each group were individually marked with picric acid and bled from the retro-orbital plexus after the second immunization, and the sera stored. Individual animal sera were analyzed via ELISA for the determination of antibody titers regarding total IgG. ELISA plates were coated with 250 ng of rChi2, blocked with PBS-T plus 10% non-fat dry milk, and incubated with sera (1:800), previously adsorbed to *E. coli* cell extract, from immunized and control hamsters. The plates were then washed and incubated with HRP-conjugated anti-hamster IgG (1:10,000, Sigma). After six washing steps, reactivity was determined as described previously.

### 2.8. Anti-rChi2 Antiserum Reactivity against Different Leptospiral Species

Pathogenic *L. interrogans*, *L. santarosai, L. kirschneri, L. borgpetersenii*, and *L. noguchii* and the saprophyte *L. biflexa* were recovered from the culture medium via centrifugation, washed twice, and then, resuspended in PBS to an OD_420nm_ equal to 0.1, and 100 µL of each were coated onto ELISA plates for 16 h at room temperature. The plates were then washed with PBS and blocked with PBS containing 3% bovine serum albumin (BSA) for 2 h at 30 °C. Blocking buffer was discarded and immobilized leptospiral cells were allowed to interact with hamsters’ anti-rChi2 antisera (1:400) for 1 h. Next, plates were washed 3 times, and bound antibodies were detected using HRP-conjugated anti-hamster IgG (1:10,000, Sigma) diluted in PBS-BSA. Reactivity was revealed as described above.

### 2.9. Ethical Statements

All animal studies were approved by the Ethics Committee for Animal Research of the School of Veterinary Medicine and Animal Science, University of Sao Paulo (CEUA/FMVZ), Sao Paulo, SP, Brazil under protocol CEUA 7458030522, approved in a meeting on 11 August 2022. The Committees in Animal Research adopt the guidelines of the Brazilian College of Animal Experimentation. This work was evaluated by the Ethical Committee in Research—Brazil Platform, Ministry of Health, and presented with a Certificate of Ethical Appreciation under approval code 78770117.0.0000.8098, approved on 25 August 2020; this certifies that the work does not need to be evaluated by the Human Ethical Committee (CONEP).

## 3. Results

### 3.1. Chimeric Protein Composition and Epitope Conservation among Leptospira *spp.*

**Figure 1:**
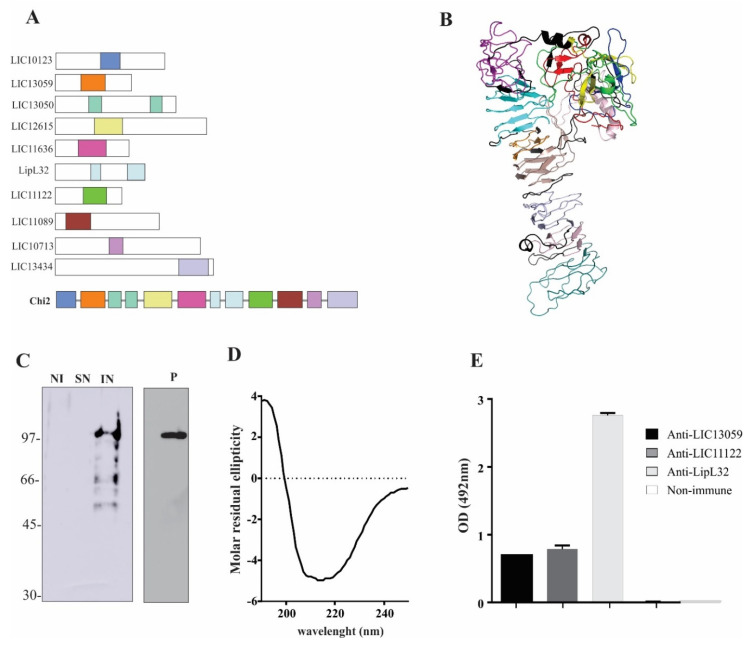
Chimeric protein design, expression, and characterization. Diverse regions of 10 conserved outer membrane proteins from *L. interrogans* were selected based on conserved domains and epitope prediction (**A**). The final amino acid sequence was used for protein modeling, indicating independent folding of each region that was interspaced by polypeptide linkers (**B**). Recombinant plasmid containing the chimeric protein coding sequence was used to transform *E. coli* BL21 (DE3) strain, and protein expression was induced by IPTG. After cell lysis, supernatant (SN) and pellet after 8M urea solubilization (IN) were evaluated via immunoblotting and probing with monoclonal anti-His antibodies (**C**). Recombinant protein was purified via metal-chelating chromatography from the insoluble fraction and reassessed via immunoblotting. Secondary structure content was evaluated via circular dichroism (**D**) and ELISA experiments with immobilized rChi2 demonstrated that the chimeric protein could be recognized by mouse hyperimmune serum against some of the proteins from which the portions were selected (**E**).

Conserved surface-exposed proteins identified using the subproteomic approach [22] were selected for the composition of the chimeric gene. The cited authors performed comparative surface-exposed proteome analysis among three representative *L. interrogans* vaccine strains (serovars Lai, Canicola, and Hebdomadis) and identified ten core antigens of interest. The corresponding *L. interrogans* serovar Copenhageni coding sequences (CDSs) were selected: LIC10123, LIC13059, LIC13050, LIC12615, LIC11636, LipL32, LIC11122, LIC11089, LIC10713, and LIC13434. A summary of the portions selected with each protein, with the number of predicted epitopes, are presented in Table 1, while the conservation of each portion of the chimeric protein among different species of *Leptospira* is shown in Table 2. When conserved domains were found within each sequence, epitope selection was restricted to those portions. The chimeric protein, termed Chi2, was designed by combining each portion interspaced by a GGGGSGGGGSGGGGS linker, and the final design is depicted in Figure 1A. The predicted tertiary structure was defined by I-TASSER and suggests independent folding of each portion (Figure 1B).

### 3.2. Expression and Purification of rChi2

The optimized conditions for expression of the chimeric protein were induction with 1 mM IPTG for 16 h at 18 °C, with constant agitation. When expression was assayed at 37 °C, the majority of the bands detected by monoclonal anti-His antibodies were degradation products (data not shown). By using the optimized protocol, the recombinant protein (rChi2) was expressed in the bacterial pellet in its insoluble form as inclusion bodies, as demonstrated by Western blotting probing with anti-His antibodies (Figure 1C, lane IN). It is worth mentioning that attempts to obtain the protein in its soluble form failed. Protein was recovered from inclusion bodies after treatment with 8 M urea. Purification was performed via metal-chelating chromatography after refolding. The integrity of the purified recombinant protein was confirmed by anti-His antibodies (Figure 1C, lane P). rChi2 protein migrated as a 100-kDa band, comprising the fusion vector plus the encoded amino acid sequence. The secondary structure content of rChi2 was assessed via CD spectroscopy (Figure 1D). The CD spectrum data analysis using BestSel software showed a combination of β-strand and alpha-helix contents of 27 and 29%, respectively, which is compatible with the predicted tertiary structure. By performing ELISA experiments with immobilized rChi2, we demonstrated that the chimeric protein could be recognized by anti-LIC13059, anti-LIC11122, and anti-LipL32 mouse hyperimmune serum available in our laboratory, denoting that the in silico selected epitopes are valid and exposed in protein folding (Figure 1E).

### 3.3. Detection of rChi2-Reactive IgM and IgG Antibodies in Pooled Human Serum Samples

**Figure 2:**
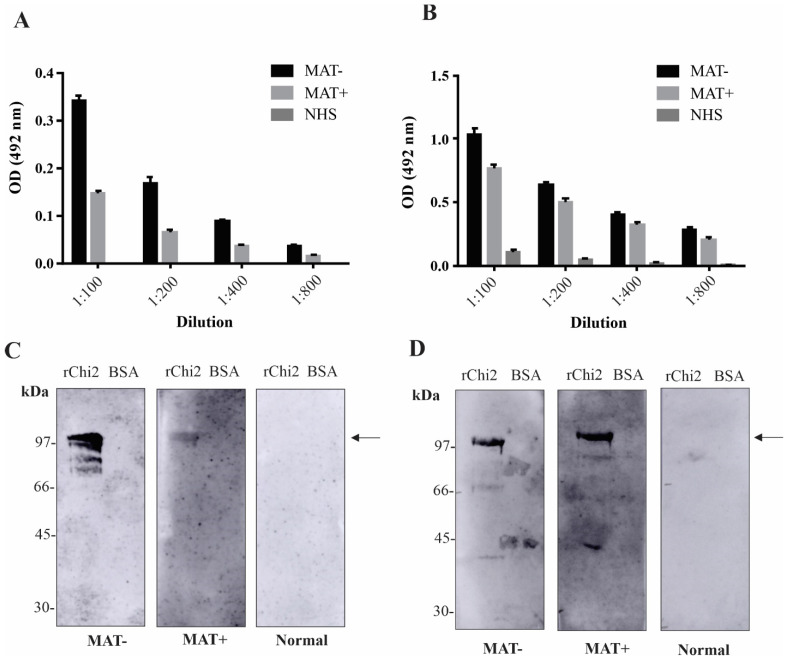
Pooled serum from patients at the onset (MAT−) and in the convalescent phase (MAT+) and normal human serum (NHS) were incubated at different dilutions (1:100 to 1:800) with the immobilized rChi2 onto ELISA plates. Reactivity was detected either with HRP-conjugated anti-human IgM (**A**) or IgG (**B**). For assessing the specificity of antigen–antibody recognition, immunoblots were performed, and transferred rChi2 or BSA (negative control) were incubated with the polled samples at 1:1000 dilution. Reactivity was detected either with HRP-conjugated anti-human IgM (**C**) or IgG (**D**).

To explore the serodiagnostic potential of rChi2, the recombinant protein was immobilized onto ELISA plates and set to interact with different dilutions of a pool of human serum samples at the onset (MAT−, n = 10) or in the convalescent (MAT+, n = 10) phase. A pool of NHS (n = 10) was employed as a control. After 1 h of interaction, the wells were incubated with either HRP-conjugated anti-human IgM or IgG. As expected, IgM antibodies were detected at higher intensity in MAT– samples (Figure 2A). No reactivity was obtained when the NHS samples were incubated with rChi2. In regard to IgG detection, strong reactivity was achieved after incubation with both MAT− and MAT+ serum samples, with only a basal sign with the NHS samples (Figure 2B). To access the specificity of the reaction, rChi2 or BSA protein was transferred to a nitrocellulose membrane, incubated with the different pools, and then, probed with HRP-conjugated anti-human IgM or IgG. As shown, the reactivity was specific for both IgM (Figure 2C) and IgG antibodies (Figure 2D), with no signal detected for the BSA band, in accordance with the signals obtained by the ELISA experiments.

### 3.4. Reactivity of rChi2 with Individual Human Serum Samples

**Figure 3:**
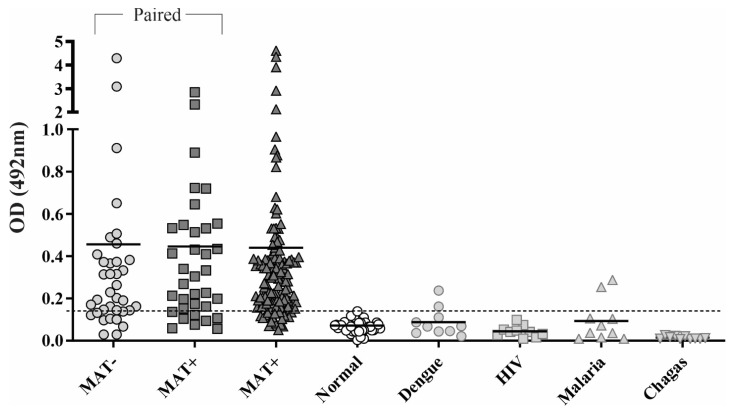
Reactivity of rChi2 with confirmed leptospirosis cases and unrelated febrile illness. The reactivity of the chimeric protein experimentally with human leptospirosis paired serum samples (n = 36) at the onset of disease (MAT-negative) and in the convalescent phase of disease (MAT-positive) and additional MAT+ samples (n = 142) were determined via ELISA. The cutoff value is shown as a dashed line, and it was defined as the mean plus 3 standard deviations obtained with NHS (n = 50). Sera from patients with unrelated febrile illness, including dengue, Chagas’ disease, HIV, and malaria (n = 10 each), were also employed for assessing the specificity of the reactions.

As both IgM and IgG antibodies were found in pooled serum samples, we proposed an ELISA experiment for the concomitant detection of IgM and IgG. Briefly, individual paired serum samples (n = 36) at the onset (MAT-negative, leptospirosis false-negatives) and in the convalescent phase (MAT-positive), and additional unpaired MAT-positive serum samples (n = 142) were incubated with immobilized rChi2, followed by incubation with HRP-conjugated anti-human IgG and IgM (1:10,000 of each antibody). For the cutoff calculation, we employed 50 NHS samples. Based on the defined cutoff (0.14), 75 and 82.5% of responders were obtained for MAT− and MAT+ samples, respectively, and as expected, all signals obtained with NHS samples remained below the cutoff value (Figure 3). Regarding the paired samples, the mean absorbance for MAT− and MAT+ was 0.45 and 0.44, respectively, in contrast to 0.07 for NHS. Unpaired MAT-positive samples displayed an average absorbance of 0.44. rChi2 proved to be a good serodiagnostic antigen with high sensitivity and prompted us to evaluate the specificity of the reaction via incubation of the recombinant protein with serum samples from patients diagnosed with unrelated illness. For HIV and Chagas’ disease sera, 100% specificity was obtained, and for dengue and malaria, 80%. However, for these patients, previous contact with *Leptospira* cannot be ruled out. These data altogether suggest that rChi2 is a promising antigen that could be used for the early detection of leptospirosis, in a timeframe where the MAT results are still negative or inconclusive.

### 3.5. rChi2 Is Immunogenic in Hamsters and Elicits Antibodies Capable of Recognizing Pathogenic Leptospira

The production of total IgG was monitored after subcutaneous immunization with rChi2 mixed with Alhydrogel adjuvant; animal sera diluted to 1:800 recognized the immobilized recombinant protein, with a mean absorbance of 0.99, contrasting with 0.08 in the saline-injected animal sera (*p* < 0.01). A discrepancy in the immunogenicity of rChi2 among animals was observed; this was consistently observed in the hamster model, since these are non-isogenic animals and their immune response may vary.

**Figure 4:**
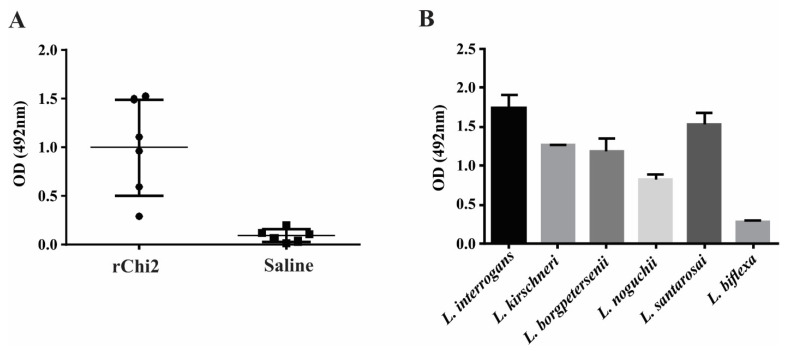
Immune response elicited via rChi2 immunization and *Leptospira* spp. cross-reaction. Hamsters (n = 6) were immunized subcutaneously with 50 µg recombinant protein mixed with 12.5% Alhydrogel on day 0 and boosted after 2 weeks. Blood was collected 2 weeks after the last immunization and sera at 1:800 dilution were utilized for the determination of antibody titers regarding total IgG via ELISA (**A**). The hamsters in the negative control group (saline) were injected with Tris-NaCl buffer in 12.5% Alhydrogel. Pooled hyperimmune antiserum was used for antigen detection on the surface of intact immobilized *Leptospira* spp. via ELISA (**B**). Saprophyte *L. biflexa* was used as a control.

Pooled sera from rChi2-immunized hamsters were incubated with different *Leptospira* species cells, and reactivity was revealed via incubation with anti-hamster IgG. As expected, pooled anti-rChi2 antisera recognized all the tested leptospiral strains. As the chimeric gene was designed on the basis of *L. interrogans* sequences, higher reactivity was obtained when antiserum was allowed to interact with immobilized *L. interrogans* cells, followed by *L. santarosai* cells, and as expected, to a lesser extent, *L. biflexa* cells (Figure 4B). These results indicate that cross-protection could be achieved through immunization with this chimeric protein.

## 4. Discussion

Leptospirosis is recognized as a globally re-emerging public health problem; when afflicting humans, the disease can be potentially fatal, affecting multiple organs such as the liver, kidney, and lungs [2,27]. Accordingly, the search for novel leptospiral antigens to improve the diagnosis of leptospirosis, particularly shortly after the presentation of symptoms, would favor early treatment and therefore improve patients’ quality of life [27].

Leptospirosis serodiagnosis remains a challenging task, whereas the WHO-recommended method for detecting anti-*Leptospira* antibodies, MAT, displays low sensitivity at the onset of symptoms [1]. This reference method is based on the agglutination of live antigen suspensions of distinct leptospiral serovars by antibodies present in the sera of suspected patients. Because of its methodological basis, MAT has its pros and cons; while it has epidemiological value, the methodology is difficult to control, perform, and interpret [28]. Furthermore, MAT presents other drawbacks such as the necessity of weekly subcultures, quality tests to check for cross-contamination within cultures, and hazards faced by laboratory technicians [2].

Several ELISA protocols based on single-OMP antigens have been developed for the serodiagnosis of leptospirosis, including the major leptospiral proteins LipL32, LipL21, LipL41, and OmpL1 [9,11,29,30,31,32,33]. In most cases, reactivities are more prominent in MAT-confirmed cases, hampering early disease confirmation. Chimeric multiepitope proteins have recently become an interesting approach to enhancing sensitivity, especially at the early stages of the disease; these proteins include LipL32-LipL21-OmpL1 [17] and rQ1, comprised of LigA-Mce-LipL41-OmpL1-Lsa45 [16] antigens. Recombinant rQ1 displayed a promising immunoprotective profile in a hamster model of infection and immunoreactivity to MAT-positive human serum samples; nonetheless, this chimeric antigen was not efficient at detecting antibodies in human serum samples at the onset of disease [16]. Some other *Leptospira* spp. chimeric constructions have been designed and explored for vaccine purposes [34,35,36,37], but their potential as a diagnostic marker has not been explored yet. Other published dry lab studies explored possible new chimeric construction by using a combination of epitope prediction and molecular docking for vaccine and/or diagnostic goals [38,39].

A new chimeric antigen was developed based on previous subproteomic results exploring conserved OMPs among *Leptospira* spp., where 10 identified conserved proteins (LIC10123, LIC13059, LIC13050, LIC12615, LIC11636, LipL32, LIC11122, LIC11089, LIC10713, and LIC13434) were evaluated regarding conserved domains and B- and T-cell epitope prediction. Some CDSs were previously characterized by our group as having full-length recombinant form via in vitro assays. These proteins, namely LIC13059 (Lsa25.6), LIC11122 (Lsa19), and LIC11089 (Lsa32), were shown to interact with host components, and a possible relevant role in bacteria–host interactions was proposed [40,41,42]. LipL32 is still considered an enigma in leptospiral biology [43], and most of the immunoprotection assays performed with the recombinant protein have rendered unsatisfactory results [44,45]. However, Maneewatch et al. [26] showed that anti-LipL32 monoclonal antibodies were able to rescue hamsters from lethal *Leptospira* infection, and the epitopes referring to those antibodies, one linear and one conformational, were mapped. Both epitopes were included in the chimeric protein. LIC10713 protein, namely LruB, has been shown to react with antibodies in sera from humans who have leptospiral uveitis [46]. LIC13434 protein has recently been characterized as a metalloprotease of the pappalysin family, capable of degrading host proteins and possibly related to leptospiral invasiveness [47].

The final antigens of 100 kDa, termed rChi2, were purified from the insoluble fraction and used for immunoassays. ELISA and immunoblotting with pooled serum from subjects at both the onset and in the convalescent phase demonstrated that the recombinant protein could be recognized by both IgM and IgG antibodies in a specific manner.

IgM antibodies are produced and detectable during the first week of illness [48,49,50], before the detection of IgG and agglutinating antibodies, and persist for at least 5 months in patients, allowing for early disease confirmation and treatment [51]. A protocol for LigA-IgM ELISA was established by Kitashoji et al. [14], but only 33% reactivity was demonstrated for MAT-negative serum samples. IgM antibodies against rChi2 were detected in MAT-negative pooled serum samples and, to a lesser extent, in MAT-positive ones. Surprisingly, high reactivity of IgG antibodies was detected in both MAT-negative and MAT-positive samples, indicating that our ELISA method could be more sensitive than the reference MAT for diagnosis at the onset of leptospirosis, since 75% of MAT-negative (leptospirosis false-negatives) samples could be distinguished as positive by rChi2 ELISA.

Supported by our observation, we established a strategy for the concomitant detection of IgM and IgG in our serum samples, to increase the sensitivity of the assay. Using paired (n = 36) and additional MAT+ (n = 142) serum samples, 75 and 82.5% of responders were obtained for MAT– and MAT+ samples; this indicates that rChi2 is a powerful antigen for early serodiagnosis, which is consistent with its immunogenicity in animal models and cross-reaction with several leptospiral species. In this work, the reactivity of human sera to the full-length chimeric protein was evaluated; combining more immunologically relevant portions within rChi2 could be an interesting issue to pursue.

The rChi2 protein elicited high antibody titers in hamsters and anti-rChi2 antisera recognize all tested leptospiral strains, despite the fact that the chimeric gene was designed on the basis of *L. interrogans* sequences. These results suggest that the immunization of this chimeric protein promoted broad cross-reactivity among *Leptospira* spp., and highlights its immunoprotective potential, which remains to be pursued.

## 5. Conclusions

We demonstrated the feasibility of a new chimeric formulation for early leptospirosis diagnosis, adding to the evidence that multiepitope antigens derived from the conserved OMPs of *Leptospira* spp. are attractive targets for future diagnostic kits. The combined use of B-cell epitopes within the 10 selected highly conserved surface proteins (LIC10123, LIC13059, LIC13050, LIC12615, LIC11636, LipL32, LIC11122, LIC11089, LIC10713, and LIC13434) has proven to be an interesting approach for detecting antibodies in human serum samples of leptospirosis.

## Figures and Tables

**Table 1:** Composition and predicted B-cell epitopes of rChi2 portions.

Protein	Conserved	Selected	Predicted
	Domain ^a^	Region	Epitopes ^b^
LIC10123	FecR	132–191	3
	(91–201)		
LIC13059	-	76–150	3
LIC13050	Big	100–140/287–324	2/2
	(67–164)/(250–343)		
LIC12615	DUF3383	120–205	4
	(270–458)		
LIC11636	AIM24	70–155	5
	(3–206)		
LipL32	LipL32	108–138/220–272	2 *
	(47–243)		
LIC11122	FecR	84–155	3
	(53–155)		
LIC11089	-	30–105	5
LIC10713	Peptidase_M75	161–203	2
	(58–351)		
LIC13434	Peptidase_M43	374–464	4
	(318–466)		

^a^: PFAM domains (http://smart.embl-heidelberg.de/ (accessed on 15 June 2015)). ^b^: Epitopes attributed to B-cells via ANTIGENIC program in EMBOSS (http://www.bioinformatics.nl/cgi-bin/emboss/antigenic (accessed on 15 June 2015)). * LipL32 epitopes attributed to monoclonal antibodies were experimentally defined by Maneewatch et al. [26].

**Table 2:** rChi2 portion conservation among pathogenic, intermediate, and saprophyte species.

	10123	13059	13050	12615	11636	LipL32	11122	11089	10713	13434
*L. interrogans* serovar Copenhageni	100	100	100/100	100	100	100/100	100	100	100	100
*L. kirschneri* Cynopteri	100	96	100/97	94	100	100/100	100	99	98	99
*L. noguchii* Panama	100	96	100/97	94	95	100/100	94	92	86	98
*L. alstoni* Pingchang	93	91	98/97	76	98	97/98	90	83	85	92
*L. weilii* Ranarum	92	89	98/97	76	98	97/98	88	82	78	90
*L. alexanderi* Manhao	95	91	100/97	ND	93	97/96	89	84	82	92
*L. borgpetersenii* Hardjo-bovis	93	89	100/97	ND	90	100/100	86	77	81	93
*L. santarosai* Shermani	93	88	100/100	40	90	100/100	89	78	81	92
*L. kmetyi* Malaysia	97	41 (c = 30)	100/95	80	95	97/96	90	69	70	88
*L. fainei* Hurstbridge	69	39	63/57	38	74	90/75	62	43	52	56
*L. broomii* Hurstbridge	69	0	60/62	44	75	85/73	62	37	48	56
*L. wolffi* Khorat	66	37 (c = 46)	70/65	ND	75	90/70	62	ND	43	56
*L. liceraciae* Varillal	65	0	64/60	ND	71	90/64	57	31	46	56
*L. inadai* Lyme	69	39	60/60	44 (c = 045)	74	86/73	62	40	48	57
*L. biflexa* Patoc	50	0	63/51	ND	ND	ND/ND	40	ND	ND	50

## Data Availability

All the data generated or analyzed during this study are included in this published article.
